# Multi-Center 3D CNN for Parkinson’s disease diagnosis and prognosis using clinical and T1-weighted MRI data

**DOI:** 10.1016/j.nicl.2025.103859

**Published:** 2025-08-05

**Authors:** Silvia Basaia, Elisabetta Sarasso, Francesco Sciancalepore, Roberta Balestrino, Simona Musicco, Stefano Pisano, Iva Stankovic, Aleksandra Tomic, Rosita De Micco, Alessandro Tessitore, Massimo Salvi, Kristen M Meiburger, Vladimir S Kostic, Filippo Molinari, Federica Agosta, Massimo Filippi

**Affiliations:** a Neuroimaging Research Unit, Division of Neuroscience, IRCCS San Raffaele Scientific Institute, Milan, Italy; b Vita-Salute San Raffaele University, Milan, Italy; c Department of Neuroscience, Rehabilitation, Ophthalmology, Genetics and Maternal Child Health, University of Genoa, Genoa, Italy; d Biolab, Polito^(BIO)^Med Lab, Department of Electronics and Telecommunications, Politecnico di Torino, Torino, Italy; e Neurorehabilitation Unit, IRCCS San Raffaele Scientific Institute, Milan, Italy; f Neurosurgery and Gamma Knife Radiosurgery Unit, IRCCS Ospedale San Raffaele, Milan, Italy; g Clinic of Neurology, Faculty of Medicine, University of Belgrade, Belgrade, Serbia; h Department of Advanced Medical and Surgical Sciences, University of Campania “Luigi Vanvitelli”, Napoli, Italy; i Neurology Unit, IRCCS San Raffaele Scientific Institute, Milan, Italy; j Neurophysiology Service, IRCCS San Raffaele Scientific Institute, Milan, Italy

**Keywords:** Parkinson’s disease, Machine learning, MRI

## Abstract

**Objective:**

Parkinson’s disease (PD) presents challenges in early diagnosis and progression prediction. Recent advancements in machine learning, particularly convolutional-neural-networks (CNNs), show promise in enhancing diagnostic accuracy and prognostic capabilities using neuroimaging data. The aims of this study were: (i) develop a 3D-CNN based on MRI to distinguish controls and PD patients and (ii) employ CNN to predict the progression of PD.

**Methods:**

Three cohorts were selected: 86 mild, 62 moderate-to-severe PD patients, and 60 controls; 14 mild-PD patients and 14 controls from Parkinson's Progression Markers Initiative database, and 38 de novo mild-PD patients and 38 controls. All participants underwent MRI scans and clinical evaluation at baseline and over 2-years. PD subjects were classified in two clusters of different progression using k-means clustering based on baseline and follow-up UDPRS-III scores. A 3D-CNN was built and tested on PD patients and controls, with binary classifications: controls *vs* moderate-to-severe PD, controls *vs* mild-PD, and two clusters of PD progression. The effect of transfer learning was also tested.

**Results:**

CNN effectively differentiated moderate-to-severe PD from controls (74% accuracy) using MRI data alone. Transfer learning significantly improved performance in distinguishing mild-PD from controls (64% accuracy). For predicting disease progression, the model achieved over 70% accuracy by combining MRI and clinical data. Brain regions most influential in the CNN’s decisions were visualized.

**Conclusions:**

CNN, integrating multimodal data and transfer learning, provides encouraging results toward early-stage classification and progression monitoring in PD. Its explainability through activation maps offers potential for clinical application in early diagnosis and personalized monitoring.

## 1 Introduction

Parkinson's disease (PD) is a complex neurodegenerative disorder characterized by diverse motor and non-motor symptoms. There is great variability in terms of disease manifestations and rate of progression, emphasizing the importance of early diagnosis and personalized treatment (Balestrino and Schapira, 2020). The diagnosis is indeed clinical and can be supported by imaging and instrumental findings; however, it may lack sensitivity, especially in early stages, underscoring the need for improved diagnostic approaches (Virameteekul et al., 2023). Moreover, PD exhibits diverse patterns of progression, and numerous attempts have been made to classify patient trajectories into more aggressive and malignant phenotypes versus milder ones (Fereshtehnejad et al., 2015). Recent studies have sought to identify prognostic factors to stratify patient risk since the prodromal phases of the disease (Berg et al., 2021). However, a definitive and effective clustering method has yet to be validated.

Advancements in medical imaging techniques, such as MRI paired with machine learning algorithms, show promise in enhancing the precision and reliability of PD diagnosis and prognosis, enabling earlier detection and stratification (Findley, 2007, Tchito Tchapga et al., 2021). Machine learning, a branch of artificial intelligence, focuses on developing algorithms that learn from data to make predictions or decisions without explicitly programming for such tasks. Convolutional neural networks (CNNs) represent a type of machine learning proficient in learning and identifying intricate patterns in data through convolution. CNN are particularly efficient at analysing complex data such as biomedical images, and can extract specific features from volumetric MRI scans. Additionally, transfer learning emerges as a method that utilized previously trained machine learning models to boost diagnostic accuracy and prognostic capabilities. This approach transfers knowledge from previously trained models to new ones, eliminating the need to create entirely new models for each MRI or clinical dataset.

Many research groups propose machine learning approaches to improve diagnosis and prognosis in PD, highlighting the relevance of robust models (Garcia Santa Cruz et al., 2023). Machine learning has been employed to differentiate PD from other parkinsonisms using different imaging techniques such as T1-weighted, T2-weighted, diffusion tensor imaging, and nuclear medicine tools (Castillo-Barnes et al., 2020, Talai et al., 2021). Additionally, CNN showed promise in PD classification using T1-weighted and neuromelanine-sensitive images (Chakraborty et al., 2020b). These studies provide valuable insights but focus on isolated aspects of PD diagnosis or prognosis. Comprehensive, clinically useful computer-aided systems require considering more than just individual proof-of-concept models.

In this context, the present study aims to develop a three-dimensional CNN based on 3D T1-weighted MRI to distinguish between PD patients and healthy subjects, as well as to predict disease progression in PD. We streamlined our model by minimizing pre-processing steps, which reduces both user burden and potential errors compared to previous approaches. An additional novel aspect of our approach relative to previous machine learning studies in PD is that multicenter reliability – and thus generalizability − of the method has been tested on three independent datasets obtained with different imaging protocols and MR scanners. This adaptability makes our model ideal for large multicenter studies where MRI images vary in acquisition protocols, offering an efficient solution for PD diagnosis and prognosis using MRI data in diverse clinical settings.

## 2 Materials and methods

### 2.1 Participants

Participants in this study were selected from three cohorts, each with its own inclusion and exclusion criteria and study protocols, as specified below (De Micco et al., 2021, Filippi et al., 2021, Filippi et al., 2020b). The criterion for inclusion in the present study was having undergone a baseline brain MRI with T1-weighted images and having been followed up with clinical evaluations for at least 2 years. For all the cohorts involved, approval was received from the local ethical standards committees on human experimentation and written informed consent was obtained from all subjects prior to study participation.

a) *Prospective cohort of mild and moderate-to-severe PD (MRI at 1.5 T).* 154 PD patients were prospectively recruited within the framework of an ongoing longitudinal project between January 2012 and December 2023 (Filippi et al., 2021, Filippi et al., 2020b). For the purpose of the present study, a sample of 60 mild and 62 moderate-to-severe PD patients was selected from the larger cohort, according to a previously published clustering analysis (Filippi et al., 2021). Baseline 3D T1-weighted images were acquired on 1.5 Tesla Philips Medical System Achieva machine. Clinical assessments at study entry, and at 1- or 2-year follow-ups were considered. Sixty controls performed baseline clinical, cognitive and MRI assessments. Inclusion/exclusion criteria, clinical and cognitive evaluations, and MRI protocol are reported in Supplementary materials. Demographic and clinical data of PD patients and controls are reported in Table 1.

**Table 1:** Demographic and clinical characteristics at study entry and at follow-up in healthy controls, mild PD and moderate-to-severe PD patients in all cohorts.

	*Mild and moderate-to-severe PD (1.5 T MRI)*			*Mild PD from PPMI database*		*De novo Mild PD (3 T MRI)*	
	Controls	Moderate-to-severe PD	Mild PD	Controls	Mild PD	Controls	Mild PD
**N**	60	62	60	14	14	38	38
**Age** **[years]**	61.79 ± 8.98 (44.90 – 82.96)	62.79 ± 7.58 (46.14 – 77.72)	73.80 ± 8.22 (39.37– 75.87)	65.80 ± 7.88 (52.50 – 75.90)	63.71 ± 10.32 (46.30 – 79.70)	62.82 ± 9.37 (41.00 – 83.00)	60.24 ± 5.67 (50.00 – 69.00)
**Sex [women/men]**	31/29	22/40	25/35	8/6	5/9	19/19	20/18
**Education** **[years]**	13.52 ± 2.57 (8 – 16)	11.42 ± 2.42 (4 – 16)	13.40 ± 2.54 (7 – 20)	17.71 ± 2.70 (12 – 22)	17.43 ± 3.25 (12 – 22)	10.85 ± 3.67 (5 – 18)	11.00 ± 4.81 (3 – 18)
**Disease duration [years]**	−	7.76 ± 4.89 (0.04 – 23.94)	1.67 ± 1.43 (0.5 – 5.69)	−	0.72 ± 0.76 (0.08 – 2.42)	−	1.21 ± 0.65 (0.33 – 3.00)
**Age at onset** **[years]**	−	54.82 ± 7.84 (41 – 76)	58.5 ± 8.15 (38 – 73)	−	65.08 ± 7.96 (51–75)	−	62.82 ± 9.37 (41–83)
**Hoehn & Yahr**	−	2.48 ± 0.58 (1 – 4)	1.02 ± 0.09 (1 – 1.5)	−	0.93 ± 0.27 (0–1)	−	1.47 ± 0.51 (1 – 2.5)
**LEDD change between baseline and 2-year follow-up**	−	191.13 ± 255.82 (−315–1100)	189.73 ± 206.29 (−140–700)	−	138.21 ± 210.27 (0–750)	−	231.24 ± 160.78 (−250–500)
**UPDRS III** **at baseline**	−	41.06 ± 12.88 (12–76)	15.90 ± 4.09 (5–23)	−	14.71 ± 5.90 (5–29)	−	18.97 ± 8.13 (7–38)
**UPDRS III** **at 2-year follow-up**	−	49.32 ± 11.23 (20–80)	25.27 ± 9.41 (7–53)	−	20.36 ± 10.12 (8–46)	−	26.55 ± 10.43 (7–51)

Values are numbers or means ± standard deviations (range). P values refer to ANOVA models, followed by post-hoc pairwise comparisons (Bonferroni-corrected for multiple comparisons), or Chi-square test. *Abbreviations*: CDR= Clinical Dementia Rating Scale; HC = healthy controls; LEDD = levodopa equivalent daily-dose; MMSE = Mini Mental State Examination; N= Number; PD = Parkinson's Disease; UPDRS = Unified Parkinson's Disease Rating Scale.
b) *Mild PD from PPMI database.* The Parkinson's Progression Markers Initiative (PPMI) database is commonly used by researchers to identify biomarkers of PD progression and diagnosis at its earlier stage. PPMI is an initiative that provides huge data containing the leading collection of biological, clinical, and imaging samples of differing modalities. For up-to-date information, see www.ppmi-info.org/. Subjects included in the present study had to have a baseline 3DT1-weighted images and at least one follow-up at 2 year. A total of 14 mild PD patients and 14 age- and sex-matched healthy controls were considered (Table 1). Details about the PPMI MRI data acquisition protocol can be seen on the official PPMI webpage.
c) *Prospective cohort of de novo mild PD patients (MRI scans at 3 T).* Patients were prospectively recruited at the Movement Disorders Unit of the First Division of Neurology at the University of Campania “Luigi Vanvitelli”, Naples, Italy between January 2013 and June 2017 (De Micco et al., 2021). Thirty-eight de novo PD patients were included at the time of clinical diagnosis of idiopathic PD (Table 1). Patients were collected with cognitive and clinical assessments and MRI scans at study entry, 1-year or 2-year follow-ups. Thirty-eight age- and sex- matched controls were also recruited. Baseline 3DT1-weighted images were acquired on the General Electric (GE) 3 Tesla MRI scanner. Detailed description of inclusion/exclusion criteria, clinical and cognitive evaluations, and MRI protocol are reported in Supplementary materials.

Participants in the current study were also included in previous studies (De Micco et al., 2021, Filippi et al., 2021, Filippi et al., 2020a).

No patients were excluded, and no cases of Parkinson’s plus syndromes were identified during 1-year or 2-year follow-ups.

### 2.2 MRI analysis

An experienced observer, blinded to patients’ identity, performed the MRI analysis. MRI analysis and CNN procedures were performed on a Dell PowerEdge T630 Linux, including high-performance GPU NVIDIA Tesla K40, with 2880 CUDA cores and High Frequency Intel Xeon E5-2623 v3 with 78 GB memory overall. Images were analyzed by two readers (SB 10 years of experience and FS 1 year of experience in neuroimaging).

To minimize human intervention, we directly input the 3D T1-weighted images from all three datasets into the CNN without any pre-processing steps.

#### 2.2.1 Convolutional neural networks

**Fig. 1:**
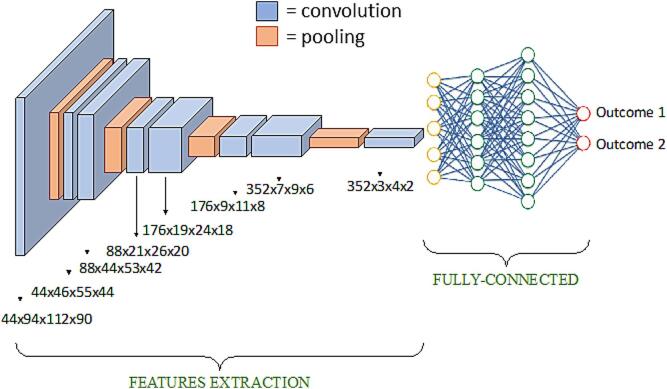
**Model architecture.** The features extraction process consists of convolutions and pooling processes to extract relevant features from input MRI volume, the fully connected performs the classification based on the previously extracted features. Blue boxes represent the convolutions. Per each convolution block, the feature map's shape is reported. Orange blocks represent the pooling operation, which consists of convolution stride set as 2. Alternating feature increase and pooling operations are implemented. In the fully connected architecture: yellow circles represent the flattened features, with possibly concatenation of metadata; green circles represent hidden neurons, 1000 elements; red circles represent the model output with softmax activation function.

Here, we introduce in detail the CNN implemented in our study **(**Fig. 1**)** (Basaia et al., 2019). First, given the volumetric nature of MR images, a network architecture that uses 3D convolutions has been developed. The inputs were 3D T1-weighted images from different cohorts and the outputs to be predicted were subject groups. The architecture of the network contains: 8 repeated blocks of convolutional layers (8 blocks of size 3 x 3 x 3 with alternating strides 1 and 2); a Rectified Linear Unit (activation layer); a fully-connected layer; and one output (logistic regression) layer. The network used in our study differs from the standard CNNs as max-pooling layers were replaced by standard convolutional layers with stride of 2 (‘all convolutional network’ (Springenberg et al., 2015)). The all convolutional network is a basic architecture reaching good performance without the need for complicated activation functions, any response normalization or max-pooling (Springenberg et al., 2015). All software was written in Python using Theano, a scientific computing library with support for machine learning and GPU computing. To supply clinical variables to the model, the data were appended to the resulting vector after flattening. This augmented vector was then inserted into the fully connected layer. Additional details concerning the CNN are reported in Supplementary materials.

To improve the performance of our classifier, we applied a technique known as transfer learning. This involved transferring the weights of the CNN used for classifying controls *vs* moderate-to-severe PD subjects to the other experiments, where they served as pre-trained initial weights (Hosseini-Asl et al., 2018). All convolutional and fully connected layers were first initialized with the weights obtained in experiment controls *vs* moderate-to-severe PD and then updated by gradient descent. “Transferring” the learned features reduces training time and enhances network efficiency. This process is crucial because the distinctive features between controls and PD may be more subtle and challenging to detect in the early stages of the disease, potentially resulting in lower accuracy in the testing set.

#### 2.2.2 CNN comparisons

Performance of the 3D CNN was validated and tested on PD patients and controls, with different binary classifications: controls *vs* moderate-to-severe PD, controls *vs* mild PD, and two cluster of PD progression. For each binary classification, different trials have been tested using 3DT1-weighted images as input into the CNN, along with demographic data (age and sex). In the comparison between different clusters of PD, the additional values of disease duration and UPDRS-III scores were also assessed.

*Controls vs moderate-to-severe PD*. 62 moderate-to-severe PD and 60 controls from cohort *a)* were inserted in the CNN (Supplementary Table 1). Experiments integrating different preprocessing strategies, including spatial normalization, intensity normalization (Min-Max scaling, Z-score, and Nyul), and brain extraction (BET) were conducted in order to assess their impact on model performance.

*Controls vs mild PD.* For this experiment, mild PD patients from cohort *a)* and all patients from cohorts *b)* and *c)* were involved. Considering healthy controls, the total number from the three cohorts was 112. In order to avoid unbalanced classes, the same amount of mild PD and controls was considered per each cohort. Overall, 224 participants were involved in this comparison, including 112 mild PD patients and 112 controls (Supplementary Table 2).

*Clusters of PD progression.* Here, we considered PD subjects from the three cohorts. In order to divide PD patients according to disease progression over time, a clustering analysis was used. Cluster analysis based on k-medoids method for data partitioning was applied using the Gower distance calculated for baseline data on demographic/general clinical information (age, age at onset, and disease duration), motor symptoms/signs (Unified Parkinson’s Disease Rating Scale [UPDRS] III total at baseline and at 2-year follow-up), levodopa equivalent daily-dose (LEDD) change between baseline and 2-year follow-up. According to the clustering, the highest silhouette index was estimated with respect of two identified PD clusters (Supplementary Table 3**)**. To assess clustering performance, the inertia measure was adopted, which computes the average distance between data points within the same cluster. Lower inertia values indicate more tightly cohesive clusters. Notably, UPDRS III scores at baseline and at 2-year follow-up emerged as the two features with the lowest inertia values, thus as the features selected to distinguish PD patients according to disease progression. Thus, patients were dichotomized into “slow” *vs*. “rapid” progressors based on their trajectory in motor scores over two years.

Each comparison included three steps (Supplementary Table 4): (i) training, (ii) validation, and (iii) testing. First, data of each classification dataset was randomly split into a large training and validation set (90% of images) and a testing set (10% of images). Data augmentation was then applied on images selected for training and validation (not testing) in order to generate additional artificial images and consequently prevent overfitting, which can occur when a fully connected layer occupies most of the parameters. Providing a CNN with more training and validation examples can reduce overfitting. Data augmentation strategy consisted of deformation, flipping, scaling, cropping and rotation of images. The augmented dataset was constructed from half of the original images, to which each transformation is applied with a 50% probability, resulting in various combinations of these transformations enhancing the variability and robustness of the dataset.

CNN performance was evaluated by several performance measures, i.e. sensitivity, specificity and accuracy. Sensitivity measures the proportion of true positives correctly identified, whereas specificity refers to the proportion of true negatives correctly identified. The accuracy of a classifier represents the overall proportion of correct classifications.

#### 2.2.3 Class activation maps

Class activation maps (CAM) were computed to highlight the most discriminative regions in the image for each subject (PD patients or controls). As described by Zhou et al. (2016) (Zhou et al., 2016), we began by performing forward propagation on the input image, obtaining the weights (w1, w2, w3… wn) from the output layer for the specific class of interest. Next, we took the feature maps (f1, f2, f3… fn) from the final convolutional layer and upsampled them to the same resolution as the original input image. To create the class activation map, we multiplied these upsampled feature maps by the corresponding weights for the class and then combined the results.

## 3 Results

Table 2, Table 3 report binary classification performances (accuracy, sensitivity and specificity) of the CNN in the training, validation and testing datasets. Supplementary Table 4 contains the dimensionality of all datasets in each trial of different comparisons (with or without data augmentation). Supplementary Table 5 reports balanced accuracy in all the comparisons.

**Table 2:** Trial performance of the CNNs in the comparison between controls and moderate-to-severe/mild PD patients.

TRIALS	ACCURACY (%)			SENSITIVITY (%)			SPECIFICITY (%)		
	Training	Validation	Test	Training	Validation	Test	Training	Validation	Test
*Controls vs moderate-to-severe PD patients*									
**T1-w**	100.00	64.09	74.04	100.00	64.40	85.76	100.00	65.54	71.31
**T1-w + age + sex**	83.17	63.31	69.75	90.02	63.08	86.29	81.91	65.62	65.65
**T1-w +** **Data augmentation**	85.93	56.12	71.20	85.05	61.66	74.67	88.11	51.74	70.56

*Controls vs mild PD patients*									
**T1-w**	56.48	51.05	49.33	56.14	59.10	43.81	57.36	44.05	53.00
**T1-w + age + sex**	56.66	49.48	50.28	56.89	57.10	45.51	57.06	43.09	54.45
**T1-w + Transfer Learning**	83.97	55.36	64.27	83.90	65.45	63.02	84.34	48.51	66.01
**T1-w + Transfer Learning + Data augmentation**	81.38	59.44	58.30	59.44	64.22	64.22	58.30	54.83	60.43

Values are presented as a percentage of the rate of the evaluation metric per dataset in each trial. *Abbreviations*: T1-w = 3DT1-weighted.

**Table 3:** Trial performance of the CNNs in the comparison between the two PD clusters of clinical progression (stable *vs* worsening).

TRIALS	ACCURACY (%)			SENSITIVITY (%)			SPECIFICITY (%)		
	Training	Validation	Test	Training	Validation	Test	Training	Validation	Test
**T1-w**	67.17	57.57	51.16	68.76	63.69	49.84	68.13	53.96	51.76
**T1-w + age + sex + disease duration + UPDRS III**	82.85	71.28	72.00	80.89	70.32	68.98	86.04	76.55	80.52
**T1-w + age + sex + disease duration**	74.29	60.77	58.05	72.68	63.08	57.48	78.21	62.82	62.01
**T1-w + Transfer Learning**	83.85	67.27	65.62	83.60	68.40	63.30	84.50	63.35	70.75
**T1-w + Transfer Learning + Data augmentation**	83.74	72.50	64.79	82.82	69.32	62.31	85.04	78.33	69.73
**T1-w + Transfer Learning + age + sex + disease duration + UPDRS III**	82.71	73.50	68.62	84.38	73.20	66.95	82.96	77.23	73.78
**T1-w + Transfer Learning + age + sex + disease duration**	81.70	68.27	65.67	82.89	70.12	64.90	82.28	67.48	69.63

Values are presented as percentage of rate of the evaluation metric per dataset in each trial. *Abbreviations:* T1-w *=* 3DT1-weighted*;* UPDRS = Unified Parkinson’s Disease Rating Scale.

### 3.1 CNN comparisons

*Controls vs moderate-to-severe PD patients*. The highest accuracy was obtained in the controls *vs* moderate-to-severe PD classification test using only 3DT1-weighted image (74.04%). When demographic information (age and sex) was incorporated into the network architecture, the accuracy rates were slightly reduced but remained high (69.75%). To mitigate overfitting, we implemented data augmentation techniques in the training and validation sets. This significantly increased the number of subjects from 93 to 147 in the training set and from 17 to 26 in the validation set. The model exclusively re-trained on 3DT1-weighted data maintained a high test accuracy of 73.58% while showing slightly lower accuracies of 89.66% and 56.06% for training and validation, respectively. We performed a series of experiments integrating different preprocessing strategies, including spatial normalization, intensity normalization (Min-Max scaling, Z-score, and Nyul), and BET, both individually and in combination. However, as detailed in Supplementary Table 6, none of these configurations improved performance compared to our original pipeline, which avoided preprocessing.

*Controls vs mild PD patients.* The comparison between controls and mild PD participants yielded generally lower performance than the previous experiment, reflecting the greater challenge in distinguishing early-stage PD. When the model received only 3DT1-weighted data, the accuracy rates were 56.48% in the training set. Introducing demographic data (age and sex) into the model did not significantly alter its performance (50.28%). Incorporating transfer learning from the model of experiment *Controls vs moderate-to-severe PD* based only on T1 scans, led to the highest results in this experiment (64.27%). To mitigate overfitting, data augmentation techniques were implemented in the training and validation sets, significantly increasing the number of subjects to 255 in the training set (compared to the original 171) and 45 in the validation set (compared to the original 31). The model was exclusively re-trained using 3DT1-weighted data and transfer learning reaching 58.30% of accuracy for the test set. Especially in the validation set, implementing transfer learning, sensitivity increased more than specificity if compared to the standard approach, reaching over 60% rate.

**Fig. 2:**
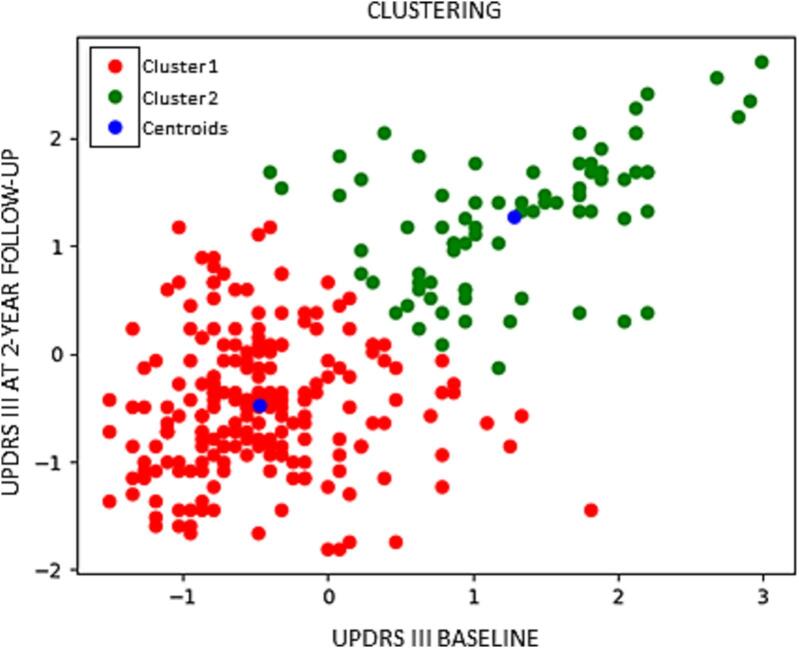
**K-means clustering based on UPDRS III assessments at baseline and follow-up.** Each dot represents a patient. Red dots denote stable PD patients, green dots denote worsening PD patients, and blue dots denote the two cluster centers. Values are standardized by removing the mean and scaling to unit variance. *Abbreviations:* PD= Parkinson’s disease; UPDRS = Unified Parkinson’s Disease Rating Scale.

*Clusters of PD progression.* According to cluster analysis, two main PD groups were identified: 207 stable (cluster 1) and 77 worsening (cluster 2) PD subjects with the latter group being older and having earlier PD onset, longer disease duration, more severe motor signs/symptoms at baseline and also a greater motor worsening over time **(**Fig. 2**)**. In order to avoid unbalanced classes, 77 stable PD were selected randomly to be age- and sex- matched with the 77 worsening PD subjects. Overall, 154 participants were involved, divided as 77 stable PD and 77 worsening PD. Demographics and clinical data of the subjects involved in this experiment are presented in Supplementary Table 3. In the comparison between *stable PD* and *worsening PD*, the proposed CNN achieved very good performance. A series of tests was conducted, varying the input data fed into the CNN. Considering 3DT1-weighted data, the accuracy rates was 51.16% in the test set. Introducing clinical (disease duration, UPDRS-III total at baseline) and demographic data (age and sex) into the model significantly improved its performance. Accuracy rates rather increased, with values of 82.85% for the training set, 71.28% for the validation set, and 72.00% for the test set. By incorporating transfer learning from the model of experiment *Controls vs moderate-to-severe PD* (based only on T1 scans), we achieved higher accuracies compared to the standard CNN: 83.85% on the training set, 67.27% on the validation set, and 65.62% on the test set. We combined transfer learning with data augmentation, increasing the number of subjects from 117 to 174 in the training set and from 21 to 31 in the validation set. Despite these enhancements, the CNN performance did not change significantly, achieving accuracies of 83.74% on the training set, 72.50% on the validation set, and 64.79% on the test set. Combining metadata and transfer learning, the performance achieved is comparable to the standard CNN with metadata implementation. In this case, the transfer learning was applied only on the convolutional layers since the connections in the last two layers were different from the experiment *Controls vs moderate-to-severe PD*, due to the inclusion of the metadata. The accuracy rate obtained was 68.62% on the validation set. To assess the influence of UPDRS III value on the classification, a trial was performed combining T1 with transfer learning, age and sex, and disease duration. On this trial, accuracy rates of 81.70%, 68.27%, and 65.67% were obtained on training, validation and test sets, respectively. If transfer learning was omitted, accuracy rates were 74.29% on training set, 60.77% on validation set and 58.05% on test set.

### 3.2 Class activation maps

**Fig. 3:**
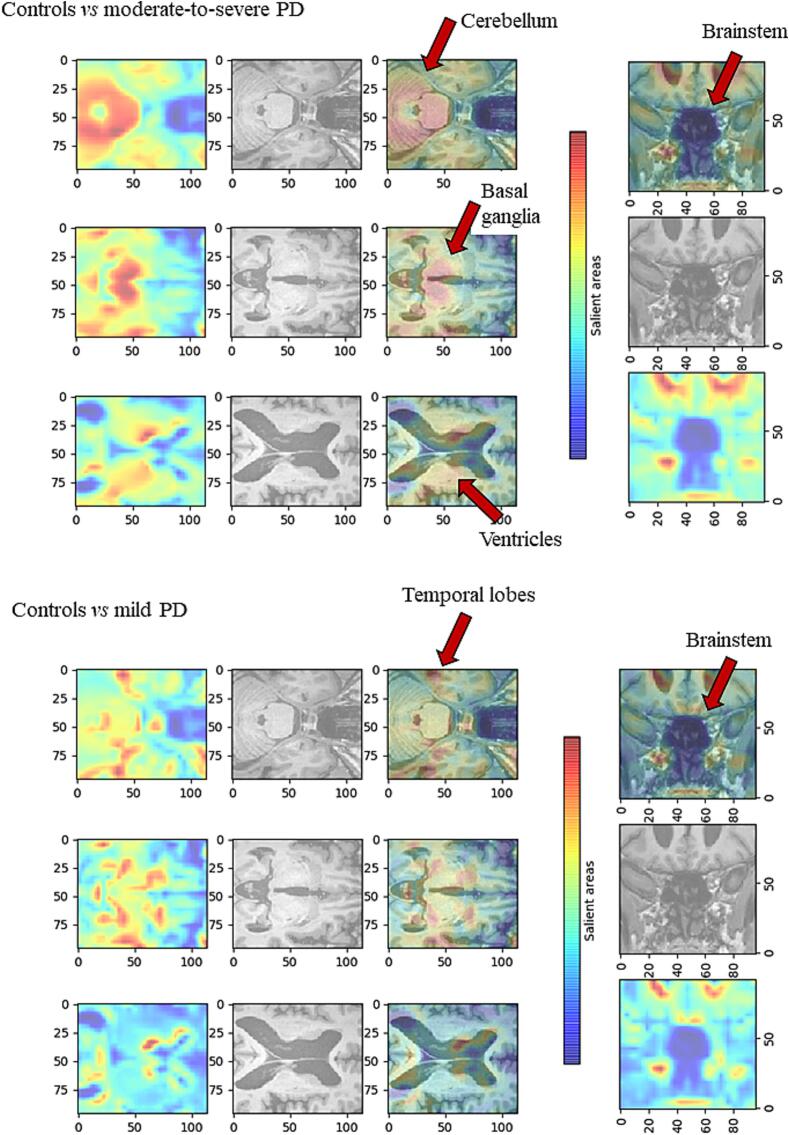
**CAM computed by the CNNs for three PD subjects involved, one for each cohort.** The rows correspond to the 1) fiftieth, 2) seventieth, and 3) ninetieth layers along the z-axis. The columns correspond to 1) the GradCAM retrieved from the CNN, 2) the input to the model, and 3) the overlap between the CAM and the MRI input. Red areas correspond to areas where critical features concerning outcome 1 (PD patients) are extracted, blue areas correspond to areas where critical features concerning outcome 0 (controls) are extracted. *Abbreviations:* PD= Parkinson’s disease.

Fig. 3 and Supplementary Fig. 1 demonstrate the CAM computed by the CNNs for three PD subjects involved, one for each cohort. In *moderate-to-severe* PD subjects compared to controls, the activations were predominantly localized within the cerebellum, basal ganglia and periventricular regions. In *mild* PD patients compared to controls, the activations were predominantly localized within temporal lobe and in the basal ganglia. The brainstem was interested in both *moderate-to-severe* PD and *mild* PD compared to controls. Finally, the CAM reported changes in the temporal lobe and basal ganglia as relevant features for differentiating cluster of different PD progression.

## 4 Discussion

The objective of this study was to assess the usefulness of machine learning in the diagnosis of PD at different stages and in the prediction of disease progression. To this aim, three experiments were conducted, as explained in the methods section, to explore the performance of CNNs based on demographic, clinical and 3DT1-weighted images in identifying: *(i)* Controls vs moderate-to-severe PD patients; *(ii)* Controls vs mild PD patients; and *(iii)* Clusters of PD progression*.* As expected, task *i)* yielded the highest performance relative to tasks *ii*) and *iii*) even without leveraging on transfer learning. Indeed, moderate-to-severe PD is characterized by greater brain alterations that are easily distinguishable from controls on MRI T1 scans (Filippi et al., 2021, Filippi et al., 2020b). Data augmentation marginally reduced overfitting but increased computational time due to the expanded dataset. In the tasks *ii)* and *iii)*, the performance was lower if compared to experiment *i)*, probably due to less evident MRI alterations in mild PD compared to controls (Filippi et al., 2021, Filippi et al., 2020b) and to the wider source of subjects as compared to task *i).*

To increase the accuracy of the model in distinguishing between mild PD patients and controls, we used transfer learning, a technique that gains knowledge from simpler and more general tasks (Sivaranjini and Sujatha, 2020). In moderate-to-severe PD patients, structural brain alterations are more pronounced and easily recognizable as compared to mild PD, allowing the CNN in discerning relevant features more effectively when classifying cases compared to controls: transferring this knowledge helps the CNN in extracting features within the relevant areas boosting the feature extraction process in harder tasks, in this case classifying mild PD. If the number of available data is limited, the model’s performance can be affected. To overcome this challenge, we implemented data augmentation strategy in order to obtain a larger dataset in the training phase. Providing the model with more inputs allows it to learn relevant patterns and increase its ability to correctly perform on unseen data. This technique involves applying different transformations to the existing real data, including flipping, cropping, scaling, and elastic deformation, resulting in a 50% augmentation of the training and validation set dimensionality across all experiments (Esmaeilzadeh et al., 2018). Recent study with PPMI data achieved high accuracy using a CNN including 2DT2-weighted MR images and transfer learning, but this approach required extensive pre-processing and a large number of images, limiting its clinical applicability (Sivaranjini and C.M.S., 2019). Although this method and other non-CNN pipelines, such as Chakraborty et al.’s multilayer perceptron on 107 hand-crafted subcortical features (95.3% accuracy) and a FreeSurfer plus Random Forest workflow reporting approximately 80–90% accuracy, rely on extensive preprocessing and cannot perform end-to-end 3D volumetric feature extraction, they underscore the practical and methodological advantages of our unified 3D-CNN for simultaneous staging and progression prediction. (Chakraborty et al., 2020a, Chudzik, 2024, Sakai and Yamada, 2019).

Furthermore, previous studies (Esmaeilzadeh et al., 2018, Sivaranjini and C.M.S., 2019) have lacked the clinical history necessary for defining disease severity, particularly in distinguishing between moderate-to-severe PD and mild PD, despite the significant impact of disease severity on the model's discriminatory capabilities. Our study stands out for addressing these challenges more comprehensively. We collected detailed clinical data that allowed us to distinguish between different disease severities and progression trajectories.

Predicting disease progression solely based on T1-weighted MRI data is a challenge for CNNs, particularly in distinguishing between stable and worsening PD cases (task *iii*). However, the integration of transfer learning brings the model's accuracy to a comparable level with that of the task *ii).* The integration of baseline UPDRS as an input feature significantly enhances the model's classification performance. Furthermore, incorporating other clinical features such as age at onset, disease duration, and LEDD improves the model's performance compared to the standard approach, albeit to a lesser extent than UPDRS inclusion alone. It is interesting to note that by removing the UPDRS III from the model (given its clustering-based nature) but adding transfer learning, the model achieves a similar accuracy to the one with all clinical data and T1 but without transfer learning.

Our aim was also to develop a model capable of generalizing across various data sources and imaging protocols, thereby enhancing its practical utility. By incorporating different cohorts and heterogeneous MRI data, our model accounts for the variability introduced by different MRI acquisition machines and protocols. Several studies have explored machine learning models for analyzing MRI data. Some have focused on using CNNs to enhance generalizability despite variations in acquisition protocols. Others have achieved accuracy through support vector machine models but requiring hand-crafted features and extensive pre-processing (Camacho et al., 2023, Salvatore et al., 2014). Additionally, some models have been proposed for predicting UPDRS with low mean absolute error, though their reliance on manually designed features may limit clinical usability (Mehrbakhsh Nilashi et al., 2017). Compared with previous literature, our approach does not involve any pre-processing technique, minimizing the burden for the users and limiting possible errors, making this model ideal − for example − in large multicenter studies, in which MRI images are acquired on different machines with different protocols and would have to be preprocessed either centrally or in each center, further introducing heterogeneity and risk of errors. Furthermore, in our model the implemented architecture is a simple CNN that consists of convolutional layers operating on MRI volumes. Increasing the complexity of the model or relying on more inputs (merging different MRI sequences) may result in a more robust and accurate tool but requires longer training and more powerful hardware making it less practical for clinical use (Soheil Esmaeilzadeh and Adeli, 2018).

Machine learning models are often described as a black box due to their intrinsic lack of interpretability, making it challenging to comprehend the decision-making process and customize the model’s architecture or input to adjust errors. To address this significant limitation, we introduced GradCAMs (Zhou et al., 2016). Through this approach, it was possible to identify the specific area of the input from which the model extracts the most relevant features, enabling a better understanding of the affected brain areas and facilitating the exploration of pathology spread. CAMs from PD at different stages allow to understand the progression of the disease. According to the Braak staging for PD (Braak et al., 2004), the brainstem is involved from the early stages, with early temporal lobe atrophy noted in mild PD, potentially serving as a biomarker for cognitive decline (Yau et al., 2018, Zhou et al., 2020). Moderate-to-severe PD patients showed widespread involvement, including basal ganglia, cerebellum, and enlarged ventricles. Extensive brain abnormalities have been previously shown in advanced PD stages (Laansma et al., 2021). GradCAMs obtained in task *iii)* are similar to those obtained in task *ii)* suggesting that stable PD are comparable to controls while worsening PD patients present a further progressive alterations of those areas involved in mild PD*.* Furthermore, recent studies reported that atrophy of the midbrain and substantia nigra has been associated with a faster progression of the disease and worsening motor symptoms. Additionally, atrophy in the putamen and caudate were correlated with non-motor symptoms such as depression and autonomic dysfunction.

Even while the results are encouraging, some limitations must be considered. Limited dataset dimensions can affect performance and generalization capability. Moreover, designing simple CNN architecture can limit learning capacity since the model cannot be able to catch all the typical intricate disorder patterns. The lack of a gold standard for discriminating between stable PD and worsening PD might affect results reliability. Future experiments should apply this technique to a larger sample, to increase model’s performance. Finally, the possibility of expanding the available dataset with other MRI modalities, such as functional MRI and diffusion tensor imaging, would further improve model's performance opening up possibilities for more in-depth studies.

In summary, the application of machine learning, particularly through a multi-modal CNN, proves to be a valuable tool for exploring different stages and progression in PD. This study successfully highlights the importance of considering PD severity in disease detection, particularly within a machine learning framework. Furthermore, the study proved the effectiveness of transfer learning in enhancing the accuracy of PD classification and progression. Looking ahead, the potential for designing more complex architectures in the CNN, the increasing availability of training data, and the availability of gold-standard methods to study disease progression, suggest promising CNN implementation in the clinical setting.

## Disclosure statement

S. Basaia and E. Sarasso have received research supports from the Italian Ministry of Health. F. Sciancalepore, R. Balestrino, S. Musicco, S. Pisano, I. Stankovic, A. Tomic, R. De Micco, A. Tessitore, M. Salvi, FM. Meiburger, F. Molinari and V.S. Kostic report no disclosures relevant to the manuscript. F. Agosta is Associate Editor of NeuroImage: Clinical, has received speaker honoraria from Biogen Idec, Italfarmaco, Roche, Zambon and Eli Lilly, and receives or has received research supports from the Italian Ministry of Health, the Italian Ministry of University and Research, AriSLA (Fondazione Italiana di Ricerca per la SLA), the European Research Council, the EU Joint Programme – Neurodegenerative Disease Research (JPND), and Foundation Research on Alzheimer Disease (France). M. Filippi is Editor-in-Chief of the Journal of Neurology, Associate Editor of Human Brain Mapping, Neurological Sciences, and Radiology; received compensation for consulting services from Alexion, Almirall, Biogen, Merck, Novartis, Roche, Sanofi; speaking activities from Bayer, Biogen, Celgene, Chiesi Italia SpA, Eli Lilly, Genzyme, Janssen, Merck-Serono, Neopharmed Gentili, Novartis, Novo Nordisk, Roche, Sanofi, Takeda, and TEVA; participation in Advisory Boards for Alexion, Biogen, Bristol-Myers Squibb, Merck, Novartis, Roche, Sanofi, Sanofi-Aventis, Sanofi-Genzyme, Takeda; scientific direction of educational events for Biogen, Merck, Roche, Celgene, Bristol-Myers Squibb, Lilly, Novartis, Sanofi-Genzyme; he receives research support from Biogen Idec, Merck-Serono, Novartis, Roche, the Italian Ministry of Health, the Italian Ministry of University and Research, and Fondazione Italiana Sclerosi Multipla.

## CRediT authorship contribution statement

**Silvia Basaia:** Writing – review & editing, Writing – original draft, Formal analysis, Data curation, Conceptualization. **Elisabetta Sarasso:** Writing – review & editing, Data curation. **Francesco Sciancalepore:** Writing – review & editing, Writing – original draft, Data curation, Conceptualization. **Roberta Balestrino:** Writing – review & editing, Data curation. **Simona Musicco:** Writing – review & editing, Methodology, Data curation. **Stefano Pisano:** Writing – review & editing, Formal analysis, Data curation. **Iva Stankovic:** Writing – review & editing, Data curation. **Aleksandra Tomic:** Writing – review & editing, Data curation. **Rosita De Micco:** Writing – review & editing, Conceptualization. **Alessandro Tessitore:** Writing – review & editing, Conceptualization. **Massimo Salvi:** Writing – review & editing, Conceptualization. **Kristen M Meiburger:** Writing – review & editing, Conceptualization. **Vladimir S Kostic:** Writing – review & editing, Funding acquisition. **Filippo Molinari:** Writing – review & editing, Conceptualization. **Federica Agosta:** Writing – review & editing, Writing – original draft, Supervision, Funding acquisition, Data curation, Conceptualization. **Massimo Filippi:** Writing – review & editing, Writing – original draft, Supervision, Funding acquisition, Data curation, Conceptualization.

## Funding

This research was supported by grants from Italian Ministry of Health (GR-2018-12366005, RF-2018-12366746, GR-2021-12374601).

## Declaration of Competing Interest

The authors declare the following financial interests/personal relationships which may be considered as potential competing interests: Massimo Filippi reports financial support was provided by Ministry of Health. Silvia Basaia reports financial support was provided by Ministry of Health. Elisabetta Sarasso reports financial support was provided by Ministry of Health. Silvia Basaia reports a relationship with Ministero della Salute that includes: funding grants. Elisabetta Sarasso reports a relationship with Ministero della Salute that includes: funding grants. Federica Agosta reports a relationship with Biogen Idec, Italfarmaco, Roche, Zambon and Eli Lilly that includes: funding grants and speaking and lecture fees. Massimo Filippi reports a relationship with Alexion, Almirall, Bayer, Biogen, BMS, Celgene, Chiesi Italia, Eli Lilly, Genzyme, Janssen, Merck, Neopharmed Gentili, Novartis, Novo Nordisk, Roche, Sanofi, Takeda, TEVA that includes: consulting or advisory, funding grants, and speaking and lecture fees. F. Agosta is Associate Editor of NeuroImage: Clinical; M. Filippi is Editor-in-Chief of the Journal of Neurology, Associate Editor of Human Brain Mapping, Neurological Sciences, and Radiology If there are other authors, they declare that they have no known competing financial interests or personal relationships that could have appeared to influence the work reported in this paper.

## Data Availability

Data will be made available on request.
