# Supplementary material for: Multi-Center 3D CNN for Parkinson’s disease diagnosis and prognosis using clinical and T1-weighted MRI data
Source: Neuroimage Clin. 2025 Aug 5;48:103859. doi: 10.1016/j.nicl.2025.103859 (PMC12351178; doi:10.1016/j.nicl.2025.103859)
Supplement: Supplementary Data 1 [file mmc1.docx]

**SUPPLEMENTARY MATERIALS**

**Inclusion/exclusion criteria**

*Prospective cohort of mild and moderate-to-severe PD (MRI at 1.5T).* Subjects were included in the study regardless of the age, sex, education, family history of PD, age at onset, side of onset, disease duration, and levodopa equivalent daily dose (LEDD). Patients were excluded if they had: modified Hoehn and Yahr (mH&Y) stage score >4 ([Hoehn and Yahr, 2001](#_ENREF_4)); moderate/severe head tremor at rest; dementia; cerebrovascular disorders or intracranial masses on routine MRI; history of traumatic brain injury; any other major neurological and medical condition.

*Mild PD from PPMI database.* Patients were invited to the PPMI study if they were of at least 30 years of age and were selected for study participation if they fulfilled four criteria: 1. presented two of three cardinal symptoms (bradykinesia, rigidity or resting tremor); 2. diagnosis within 2 years prior to entering the study; 3. untreated for PD on entering the study; 4. had a deficit of dopamine transporter, as assessed by 123I-ioflupane dopamine transporter Datscan imaging ([Nalls et al., 2015](#_ENREF_5)). More details about the PPMI inclusion and exclusion criteria can be seen on the official PPMI webpage (see [www.ppmi-info.org/](http://www.ppmi-info.org/)).

*Prospective cohort of de novo mild PD patients (MRI scans at 3T).* PD patients with a modified Hoehn and Yahr (mH&Y) stage ≤ 2.5 were included. Exclusion criteria were: a) PD onset before the age of 40 years; b) any previous or actual treatment with dopaminergic, anticholinergic, antidepressants or other centrally acting drugs; c) relevant cognitive impairment associated with PD; d) any other clinically significant medical condition; and e) cerebrovascular disorders on routine MRI. A group of healthy age and sex-matched controls with no familiar history of PD nor parkinsonism were also included.

**Clinical evaluations**

*Prospective cohort of mild and moderate-to-severe PD (MRI at 1.5T).* At study entry and each follow-up visit, an experienced neurologist blinded to MRI results performed clinical assessments. Patients were examined in ON state (i.e., period when the dopaminergic medication is working, and symptoms are well controlled). Demographic, general clinical and family data (sex, education, age, handedness, age at onset, side of onset, PD duration and family history) were obtained using a semi-structured interview. Levodopa equivalent daily dose (LEDD) was calculated. Disease severity was defined using the mH&Y stage score and Unified Parkinson’s Disease Rating Scale (UPDRS). UPDRS was used to evaluate non-motor symptoms (UPDRS I), motor symptoms (UPDRS II), and motor signs (UPDRS III).

At study entry and each follow-up visit, patients performed neuropsychological evaluations within 48 hours from MRI. For details on the neuropsychological tests see ([Filippi et al., 2021](#_ENREF_2); [Filippi et al., 2020](#_ENREF_3)).

*Mild PD from PPMI database.* Demographic and general clinical data (UPDRS and mH&Y) were extracted from the PPMI dataset.

*Prospective cohort of de novo mild PD patients (MRI scans at 3T).* Motor assessment was performed at baseline and at each follow-up visit in the “practical off” state (when the patient was free of dopaminergic medication overnight for a period of at least 12 hours). Disease severity and motor performance were assessed using the mH&Y stages and the UPDRS III. Global cognitive functioning was assessed in patients and controls. For details concerning neurophyscological assessment see ([De Micco et al., 2021](#_ENREF_1)).

**MRI acquisition**

*Prospective cohort of mild and moderate-to-severe PD (MRI at 1.5T).* Baseline and follow-up brain MRI scans were acquired on the same 1.5 Tesla Philips Medical System Achieva machine. Subjects were scanned between 10 and 11 a.m., i.e., treated PD patients were 90-120 min after their regular morning dopaminergic therapy administration (ON state). The following MR sequences were obtained: (i) 3D sagittal T1-weighted Turbo Field Echo (TFE) (frequency direction=anterior-posterior, TR=7.1 ms, TE=3.3 ms, inversion time= 1000 ms, flip angle=8°, matrix size=256×256×180 [inferior-superior, anterior-posterior], FOV=256×256mm^2^ , section thickness= 1 mm; voxel size= 1 × 1 × 1 mm, out-of-plane SENSE parallel reduction factor= 1.5, sagittal orientation). In healthy controls, resting state (RS) functional MRI (fMRI) was also obtained: gradient-echo (GRE) echo planar imaging (EPI) for (TR=3000 ms, TE=35 ms, flip angle=90°, matrix size=128×128, FOV=240×240 mm^2^; slice thickness=4 mm, 200 sets of 30 contiguous axial slices). During RS-fMRI scanning, subjects were instructed to remain motionless, to keep their eyes closed, and not to think about anything in particular.

*Mild PD from PPMI database.* PPMI’s T1-weighted MR images were selected for this study. These images were generated by using a 1.5–3 Tesla scanner. The total scan time is in the range between 20–30 min. The T1-weighted MR images were acquired as a 3D sequence with a slice thickness of 1.5 mm or less, under three different views: axial, sagittal and coronal.

*Prospective cohort of de novo mild PD patients (MRI scans at 3T).* Baseline brain MRI scans were acquired on the General Electric (GE) 3 Tesla MRI scanner equipped with an 8-channel parallel head coil. The following MR sequence was obtained: 3D high-resolution T1-weighted sagittal images (GE sequence inversion recovery prepared fast spoiled gradient recalled (IR- FSPGR), TR = 6988 ms, inversion time (TI) = 1100 ms, echo time (TE) = 3.9ms, flip angle = 10, voxel size = 1 × 1 × 1.2 mm^3^).

**MRI analysis**

**Convolutional neural networks**

Here, we introduce in detail the CNNs implemented in our study **(Figure 1)**. First, given the volumetric nature of MR images, a network architecture that uses 3D convolutions was developed. This model included 12 convolutional layers, a fully connected layer, and a softmax layer. The input data was presented in the format (4x1x96x114x92), with the respective dimensions representing the batch dimension (4), the number of channels (1), and the size of the volumetric patch (96x114x92). The batch dimension was set to 4 in order to reduce the learning variability compatibility with the resources available. Bigger batch size requires more memory usage but reaches convergence faster, smaller batch size increases the model’s generalizability capacity but slower the training phase.

The kernels employed had a size of 3x3x3, and the stride of these kernels alternates between 1 and 2. Traditional max-pooling layers were substituted with standard convolutional layers with a stride of 2. The initial number of kernels was set at 44, and this number alternates by multiplying it by either 1 or 2. The activation function for the convolutional layers was set as ReLU. The border mode for the convolutional layers was set as “valid”, with this border mode the output was smaller than the input because the convolution is only computed where the input and the filter fully overlap.After the final convolutional layer, the resulting tensor assumes dimensions of (4x352x7x9x6). This tensor is then flattened in an 8448 ($352 x 3 x 4 x 2=8448$) elements vector, and directed into a fully connected architecture comprising 1000 hidden elements. A dropout probability of 0.3 was applied to these hidden elements. The activation function for the hidden elements was set as ReLU. The activation function for the fully connected layer was softmax, with an additional dropout probability of 0.3.

To supply metadata to the model, the data were appended to the resulting vector after flattening. This augmented vector was then passed into the fully connected layer.

During the training phase, the model was trained using batches of 4 samples from the training set. The loss, which measures the difference between the model's output and the desired output, was computed for each batch. The loss function was fine-tuned using L1 and L2 regularization. L1 regularization introduces a term that is directly proportional to the absolute sum of the weights in the last two layers of the model (layers 9 and 10). Similarly, L2 regularization introduces a term that is proportional to the sum of the squares of the weights in these layers. Both regularization terms had a proportional coefficient set to 0.0001.

The model's weights were updated based on the gradient of the loss function with respect to each parameter. The calculated gradient was further adjusted using a learning rate coefficient, which is set to 0.001. The learning rate controls the size of the steps taken during the training process, aiming to reach the minimum of the loss function while avoiding fluctuations and excessively long training times. The value of the learning rate was set after a few tests where it was the best compromise.

The training process consists of 200 epochs, where the complete training set was presented to the model 200 times. After each epoch, the model's performance was assessed using the validation set. The validation set serves as a valuable indicator of the model's ability to generalize its learned knowledge to new unseen data. Following the completion of the 200 epochs, the model was finally evaluated using the test set. This evaluation assesses the model's capability to accurately perform on previously unseen data, serving as a marker of its real-world generalization potential. In the comparison between controls and moderate-to-severe PD patients, we implemented an explicit early-stopping rule to prevent overfitting by replaying every training trial. After a compulsory warm-up of 100 epochs to ensure sufficient learning, the loop terminates whenever the validation loss fails to improve by at least 0.0001 for 20 consecutive epochs. The patience counter is reset only when the criteria are satisfied. If the early-stopping condition is never met, training proceeds to the full 200 epochs; otherwise, the best checkpoint encountered before stopping is retained.

Since the distinctive features between controls and PD patients might be subtler or more challenging to detect in the early stages of the disease, the training of the model has difficulties in converging, potentially resulting in poor accuracy. To help the model discover significant features in more challenging tasks, the transfer learning method has been employed. Notably, during the initial training phase, the model was exposed to data from more advanced stages of the disease.

With this approach, the knowledge retrieved in easier and more general tasks is transferred to more specific tasks. The model parameters were initialized based on the parameters of a pre-trained model with the same architecture. Then the parameters were finetuned for this task. The learning rate was adjusted from 0.001 to 0.00001 in order the slowly find optimal weights and avoid fluctuations. The number of epochs was reduced to 150 instead of 200 because of overfitting, decreasing the number of times the training set is presented to the model will make its weights less tailor-made.

**References**

De Micco, R., Agosta, F., Basaia, S., Siciliano, M., Cividini, C., Tedeschi, G., Filippi, M., Tessitore, A., 2021. Functional Connectomics and Disease Progression in Drug-Naive Parkinson's Disease Patients. Mov Disord 36, 1603-1616.

Filippi, M., Basaia, S., Sarasso, E., Stojkovic, T., Stankovic, I., Fontana, A., Tomic, A., Piramide, N., Stefanova, E., Markovic, V., Kostic, V.S., Agosta, F., 2021. Longitudinal brain connectivity changes and clinical evolution in Parkinson's disease. Mol Psychiatry 26, 5429-5440.

Filippi, M., Sarasso, E., Piramide, N., Stojkovic, T., Stankovic, I., Basaia, S., Fontana, A., Tomic, A., Markovic, V., Stefanova, E., Kostic, V.S., Agosta, F., 2020. Progressive brain atrophy and clinical evolution in Parkinson's disease. Neuroimage Clin 28, 102374.

Hoehn, M., Yahr, M., 2001. Hoehn MM, Yahr MD. Parkinsonism: onset, progression and mortality. Neurology 17: 427-442. Neurology 57, S11-26.

Nalls, M.A., McLean, C.Y., Rick, J., Eberly, S., Hutten, S.J., Gwinn, K., Sutherland, M., Martinez, M., Heutink, P., Williams, N.M., Hardy, J., Gasser, T., Brice, A., Price, T.R., Nicolas, A., Keller, M.F., Molony, C., Gibbs, J.R., Chen-Plotkin, A., Suh, E., Letson, C., Fiandaca, M.S., Mapstone, M., Federoff, H.J., Noyce, A.J., Morris, H., Van Deerlin, V.M., Weintraub, D., Zabetian, C., Hernandez, D.G., Lesage, S., Mullins, M., Conley, E.D., Northover, C.A., Frasier, M., Marek, K., Day-Williams, A.G., Stone, D.J., Ioannidis, J.P., Singleton, A.B., Parkinson's Disease Biomarkers, P., Parkinson's Progression Marker Initiative, i., 2015. Diagnosis of Parkinson's disease on the basis of clinical and genetic classification: a population-based modelling study. Lancet Neurol 14, 1002-1009.

**Supplementary Table 1**. Demographics and clinical data of the subjects involved in the first comparison “*Controls vs moderate-to-severe PD”*.

|  | *Mild and moderate-to-severe PD (1.5T MRI)* | |
| --- | --- | --- |
|  | **Controls** | **Moderate-To-Severe PD** |
| **N** | 60 | 62 |
| **Age**  **[Years]** | 61.79 ± 8.98  (44.90 – 82.96) | 62.79 ± 7.58  (46.14 – 77.72) |
| **Sex [Women/Men]** | 31/29 | 22/40 |
| **Education**  **[Years]** | 13.52 ± 2.57  (8 – 16) | 11.42 ± 2.42  (4 – 16) |
| **Disease Duration [Years]** | - | 7.76 ± 7.89  (0.04 – 23.94) |
| **LEDD change between baseline and 2-year follow-up** | - | 191.13 ± 255.82  (-315 - 1100) |
| **Age at Onset**  **[Years]** | - | 54.82 ± 7.84  (41 – 76) |
| **UPDRS III**  **at baseline** | - | 41.06 ± 12.88  (12 - 76) |
| **UPDRS III**  **at follow-up** | - | 49.32 ± 11.23  (20 - 80) |

Values are reported as mean and standard deviation. *Abbreviations*: PD = Parkinson's disease.

**Supplementary Table 2**. Demographics and clinical data of the subjects involved in the second comparison “*Controls vs mild PD*”.

|  | *Mild and moderate-to-severe PD (1.5T MRI)* | | *Mild PD from PPMI database* | | *De novo mild PD (3T MRI)* | |
| --- | --- | --- | --- | --- | --- | --- |
|  | **Controls** | **Mild Pd** | **Controls** | **Mild Pd** | **Controls** | **Mild Pd** |
| **N** | 60 | 60 | 14 | 14 | 38 | 38 |
| **Age**  **[Years]** | 61.79 ± 8.98 (46.14 – 77.72) | 73.80 ± 8.22 (39.37– 75.87) | 65.80 ± 7.88  (52.50 – 75.90) | 63.71 ± 10.32  (46.30 – 79.70) | 62.82 ± 9.37  (41.00 – 83.00) | 60.24 ± 5.67  (50.00 – 69.00) |
| **Sex [Women/Men]** | 31/29 | 25/35 | 8/6 | 5/9 | 19/19 | 20/18 |
| **Education**  **[Years]** | 13.52 ± 2.57  (8 – 16) | 13.40 ± 2.54  (7 – 20) | 17.71 ± 2.70  (12 – 22) | 17.43 ± 3.25  (12 – 22) | 10.85 ± 3.67  (5 – 18) | 11.00 ± 4.81  (3 – 18) |
| **Disease Duration [Years]** | - | 1.67 ± 1.43  (0.5 – 5.69) | - | 0.72 ± 0.76  (0.08 – 2.42) | - | 1.21 ± 0.65  (0.33 – 3.00) |
| **Age at onset**  **[Years]** | - | 58.5 ± 8.15  (38 – 73) | - | 65.08 ± 7.96  (51 - 75) | - | 62.82 ± 9.37  (41 - 83) |
| **LEDD change between baseline and**  **2-Year follow-Up** | - | 189.73 ± 206.29  (-140 - 700) | - | 138.21 ± 210.27  (0 - 750) | - | 231.24 ± 160.78  (-250 - 500) |
| **UPDRS III**  **at baseline** | - | 15.90 ± 4.09  (5 - 23) | - | 14.71 ± 5.90  (5 - 29) | - | 18.97 ± 8.13  (7 - 38) |
| **UPDRS III**  **at follow-up** | - | 25.27 ± 9.41  (7 - 53) | - | 20.36 ± 10.12  (8 - 46) | - | 26.55 ± 10.43  (7 - 51) |

Values are reported as mean and standard deviation. *Abbreviations*: PD = Parkinson's disease.

**Supplementary Table 3.** Demographics and clinical data of the subjects involved in the third comparison “*Cluster of PD progression*”.

|  | **Cluster 1** | **Cluster 2** |
| --- | --- | --- |
| **N** | 77 | 77 |
| **Age**  **[Years]** | 62.49 ± 8.67  (54.40 - 91.40) | 64.10 ± 8.30  (54.40 - 91.40) |
| **Sex [Women/Men]** | 19/58 | 24/53 |
| **Education**  **[Years]** | 14.84 ± 3.45  (8 - 25) | 12.28 ± 3.03  (4 - 22) |
| **Disease Duration [Years]** | 1.29 ± 1.21  (0.5 – 6.57) | 6.49 ± 5.40  (0.16 – 23.94) |
| **Age at onset**  **[Years]** | 61.22 ± 8.79  (42.50 – 78.69) | 57.60 ± 10.26  (41 – 81.5) |
| **LEDD change between baseline and**  **2-Year follow-Up** | 229.87 ± 184.87  (0 – 1100) | 166.32 ± 217.85  (-315 – 985) |
| **UPDRS III**  **at baseline** | 17.87 ± 7.12  (5 – 41) | 40.36 ± 9.53  (19 – 62) |
| **UPDRS III**  **at 2-Year follow-Up** | 23.19 ± 8.37  (5 - 40) | 47.22 ± 8.73  (28 - 67) |

Values are reported as mean and standard deviation. *Abbreviations*: PD= Parkinson’s disease.

**Supplementary Table 4.** Dimensionality of all datasets per each comparison with or without data augmentation.

|  | **Trials without data augmentation** | | | **Trials with data augmentation** | | |
| --- | --- | --- | --- | --- | --- | --- |
|  | **Training set** | **Validation set** | **Test set** | **Training set** | **Validation set** | **Test set** |
| **Controls vs**  **moderate-to-severe PD** | 93 | 17 | 12 | 147 | 26 | 12 |
| **Controls vs**  **mild PD** | 171 | 31 | 22 | 255 | 45 | 22 |
| **Cluster 1 vs**  **Cluster 2** | 117 | 21 | 16 | 174 | 31 | 16 |

*Abbreviations*: PD= Parkinson’s Disease.

**Supplementary Table 5.** Balanced accuracy of the CNNs in all the comparisons.

| **Trials** | **Balanced accuracy (%)** | **Balanced accuracy (%)** | |
| --- | --- | --- | --- |
|  | Test | Test | |
| Controls *vs* moderate-to-severe PD patients | | Cluster 1 *vs* cluster 2 | |
| **t1-w** | 78.53% | **T1-w** | 50.83% |
| **t1-w+age+sex** | 75.97% | **T1-w+age+sex+disease duration+ UPDRS-III** | 74.75% |
| **t1-w +**  **data augmentation** | 74.88% | **T1-w+age+sex+disease duration** | 59.04% |
| Controls *vs* mild PD patients | | **T1-w +transfer learning** | 67.02% |
| **t1-w** | 48.40% | **T1-w +transfer learning + data augmentation** | 90.80% |
| **t1-w +age+sex** | 49.98% | **T1-w +transfer learning+age+sex+disease duration** | 67.27% |
| **t1-w +transfer learning** | 64.51% | **T1-w +transfer learning+age+sex+disease duration+ UPDRS-III** | 70.37% |
| **T1-w +transfer learning + data augmentation** | 57.77% |  |  |

Values are presented as a percentage of the rate of the evaluation metric per dataset in each trial. Abbreviations: T1-w=3DT1-weighted.

**Supplementary table 6.** Summary of the experiments performed under different normalization and training configurations. Each test explores variations in spatial normalization methods and training hyperparameters.

| **Experiments** | **Normalization** | | | | **Learning rate** | **BET** | **Epochs** | **TEST** | | |
| --- | --- | --- | --- | --- | --- | --- | --- | --- | --- | --- |
|  | Spatial | Min-max scaling | Z-score | Nyul |  |  |  | Sensitivity  **(%)** | Specificity  **(%)** | Accuracy  **(%)** |
| Test 1 |  | x |  |  | 0.001 |  | 200 | 51.42 | 50.70 | 51.46 |
| Test 2 |  | x |  |  | 0.000001 |  | 300 | 50.00 | 0.00 | 50.00 |
| Test 3 |  |  | x |  | 0.001 |  | 200 | 50.51 | 49.85 | 49.71 |
| Test 4 | x |  |  |  | 0.001 |  | 200 | 55.30 | 62.05 | 56.92 |
| Test 5 | x |  |  |  | 0.001 | x | 200 | 69.88 | 54.71 | 55.82 |
| Test 6 | x |  |  | x | 0.001 | x | 200 | 48.79 | 49.79 | 49.33 |
| Test 7 | x |  |  | x | 0.01 | x | 200 | 48.21 | 49.95 | 49.96 |
| Test 8 |  |  |  | x | 0.001 |  | 200 | 50.00 | 0.00 | 50.00 |
| Test 9 |  |  |  |  | 0.001 | x | 200 | 70.84 | 66.90 | 66.29 |
| Test 10 |  |  |  | x | 0.001 | x | 200 | 50.50 | 51.47 | 50.62 |

*Abbreviations*: BET = Brain Extraction Tool.


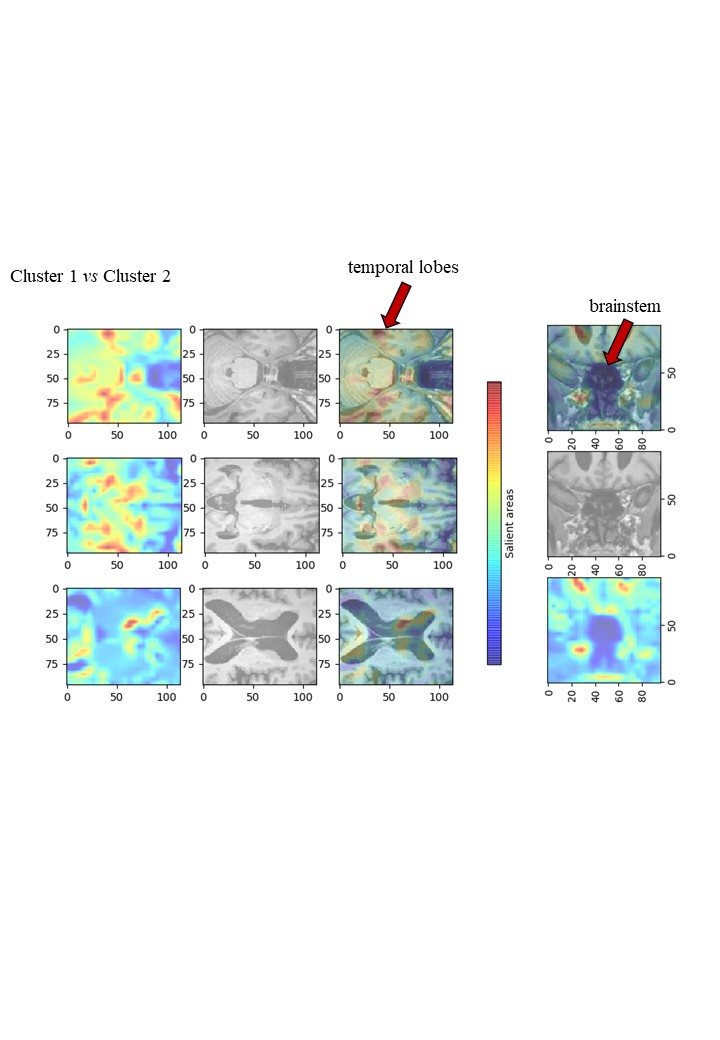


**Supplementary Figure 1.** The rows correspond to the 1) fiftieth, 2) seventieth, and 3) ninetieth layers along the z-axis. The columns correspond to 1) the GradCAM retrieved from the CNN, 2) the input to the model, and 3) the overlap between the CAM and the MRI input. Red areas correspond to areas where critical features concerning outcome 1 (cluster 2) are extracted, blue areas correspond to areas where critical features concerning outcome 0 (cluster 1) are extracted. *Abbreviations:* PD= Parkinson’s disease.
